# Supplementary material for: Subgenomic RNA and Limited Cross-Reactive Neutralising Antibodies Point to Potential Improvements in SARS-CoV-2 Clinical Handling
Source: Int J Mol Sci. 2025 Mar 24;26(7):2948. doi: 10.3390/ijms26072948 (PMC11988571; doi:10.3390/ijms26072948)
Supplement: Supplementary file 1 [file ijms-26-02948-s001.zip › Supplementary Figures.pdf]

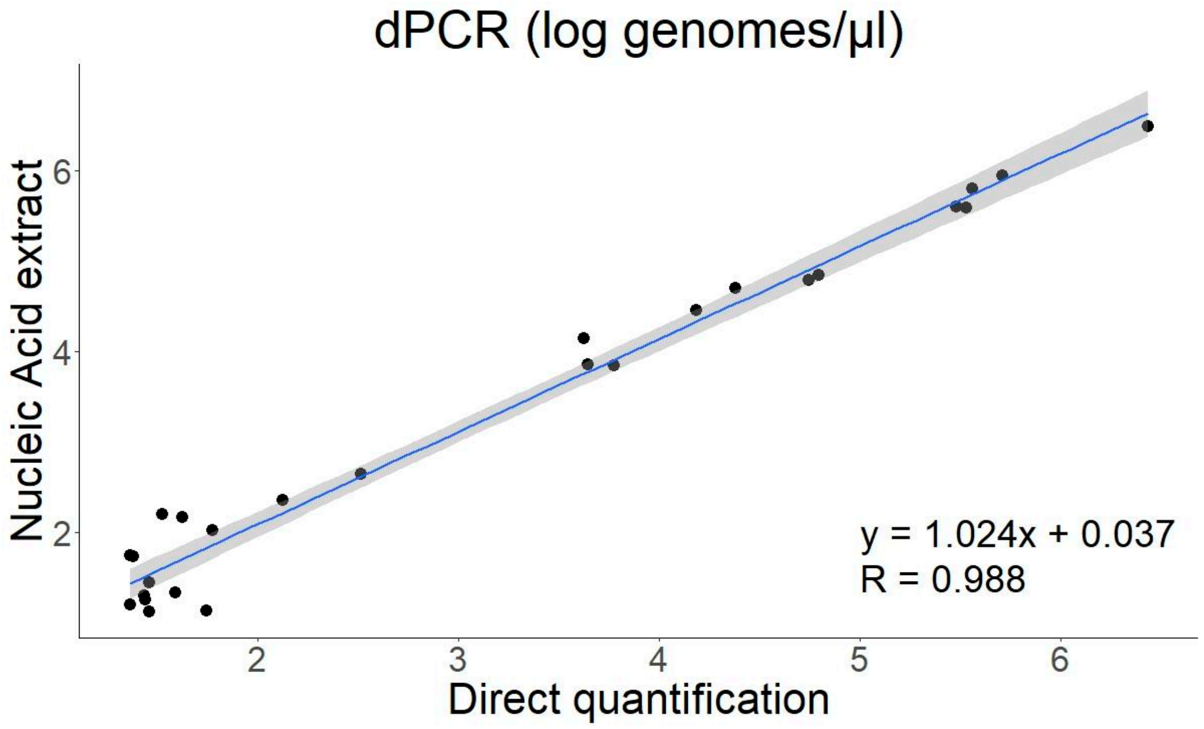

Supplementary Figure S1. dPCR can be effectively used for viral load quantification without an extraction step. In order to ensure the reliability of the direct quantification compared with the RNA extract, a subset of 27 samples was quantified using both an RNA extract and the crude sample. The comparison shows no difference in the results of the digital PCR (R^2 = 0.988), suggesting that nucleic acid extraction is an unnecessary step in SARS-CoV-2 quantification via dPCR.

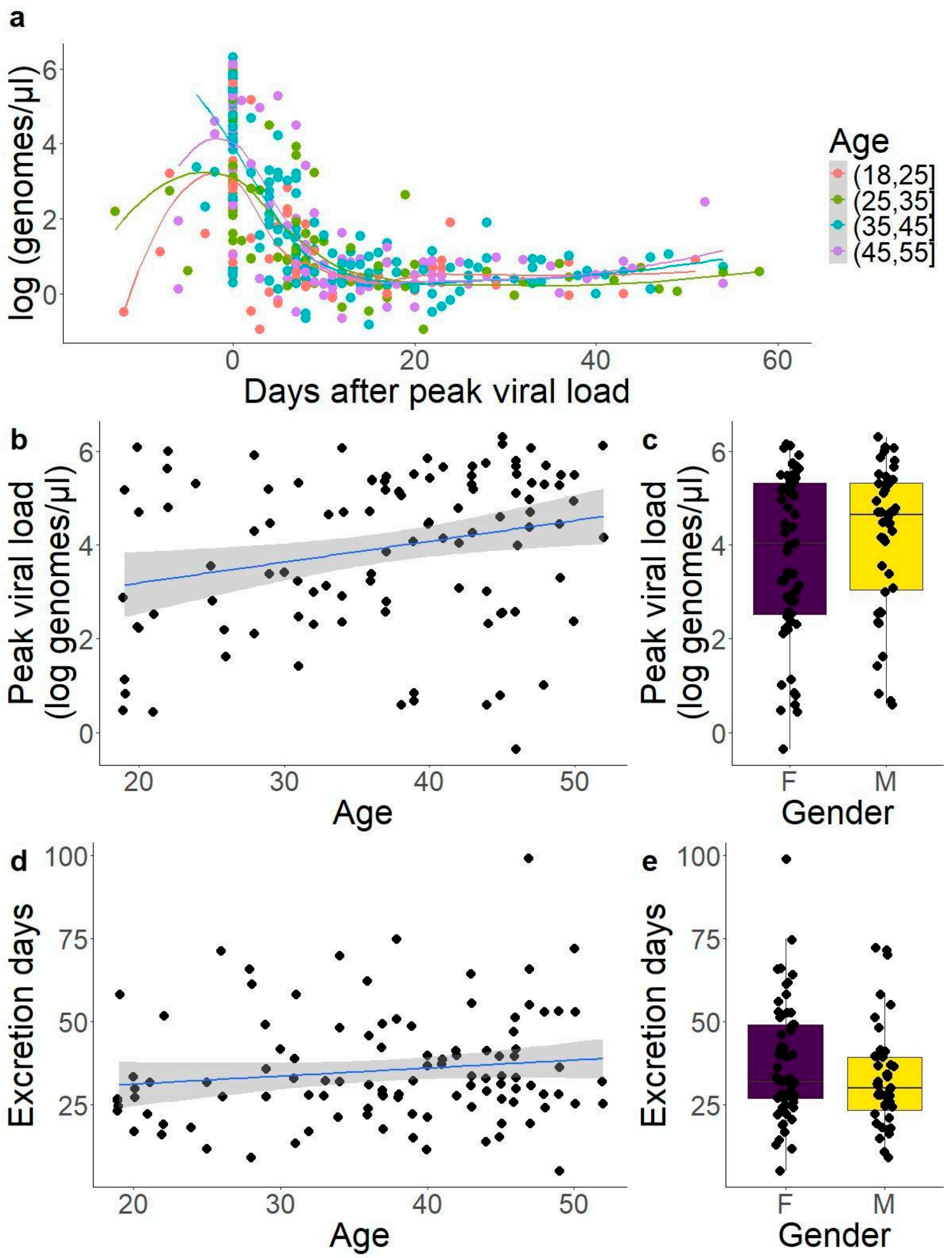

Supplementary Figure S2. Impact of age and gender in excretion dynamics. a) Excretion dynamics plot colored by age. Peak viral load (x = 0) is higher in older groups. **b)** There is a mild correlation between age and peak viral load (*p* = 0.008) **c)** Gender did not significantly impact peak viral load (*p* = 0.367). **d)** There was no statistically significant correlation between age and the number of excretion days (days with viral RNA detectable by RT-PCR) (p = 0.120)

e) Gender did not significantly impact the number of excretion days ($p = 0.194$). For b) and d), Spearman's rank correlation coefficient. The gray area represents 0.95 confidence interval. For c) and e), Wilcoxon Signed-rank test.
